# SCOPIT: sample size calculations for single-cell sequencing experiments

**DOI:** 10.1186/s12859-019-3167-9

**Published:** 2019-11-12

**Authors:** Alexander Davis, Ruli Gao, Nicholas E. Navin

**Affiliations:** 1 Department of Genetics, The University of Texas MD Anderson Cancer Center, Houston, TX USA; 2 The University of Texas MD Anderson Cancer Center UTHealth Graduate School of Biomedical Sciences, Houston, TX USA; 3 Department of Bioinformatics and Computational Biology, The University of Texas MD Anderson Cancer Center, Houston, TX USA

**Keywords:** Single cell sequencing, Sample size, Multinomial distributions

## Abstract

**Background:**

In single cell DNA and RNA sequencing experiments, the number of cells to sequence must be decided before running an experiment, and afterwards, it is necessary to decide whether sufficient cells were sampled. These questions can be addressed by calculating the probability of sampling at least a defined number of cells from each subpopulation (cell type or cancer clone).

**Results:**

We developed an interactive web application called SCOPIT (Single-Cell One-sided Probability Interactive Tool), which calculates the required probabilities using a multinomial distribution (www.navinlab.com/SCOPIT). In addition, we created an R package called pmultinom for scripting these calculations.

**Conclusions:**

Our tool for fast multinomial calculations provide a simple and intuitive procedure for prospectively planning single-cell experiments or retrospectively evaluating if sufficient numbers of cells have been sequenced. The web application can be accessed at navinlab.com/SCOPIT.

## Background

Biological tissues consist of a heterogeneous mixture of cells, including a variety of cell types in normal tissue or subclones in tumor tissue. This heterogeneity can be resolved using single-cell DNA or RNA sequencing methods [1, 2]. Single-cell sequencing studies require sufficiently many cells to be sampled so that normal cell types or cancer subclones of interest (both hereafter referred to as “subpopulations”) are represented in the sample. In most studies, however, the total number of cells is determined arbitrarily by the limits of an instrumentation run, or by budget constraints, which may result in the sampling of too few or too many cells. Here, we have developed an interactive web tool, called SCOPIT (Single-Cell One-sided Probability Interactive Tool), which provides assistance for planning experiments, using calculations from a multinomial distribution.

## Implementation

The first fact used for calculating multinomial probabilities is the well-known equivalence between the probability mass function of a multinomial distribution and conditional probabilities of a Poisson distribution. This equivalence was first noted, to our knowledge, by Fisher [3].

**Theorem 1** *Assume that*

$$ N\sim \mathrm{Multinomial}\left(p,n\right) $$

*where N and p are length k vectors, and* $\sum \limits_{i=1}^k{p}_i=1$ *. Also assume that*

$$ {X}_i\sim \mathrm{Poisson}\left({\lambda}_i\right) $$

*for i* = 1 *to k, where λ*_*i*_ = *αp*_*i*_ *for some α. Furthermore, assume that X*_1_…*X*_*k*_ *are independent. Then for any event E,*

$$ P\left(N\in E\right)=P\left(X\in E\left|\sum \limits_{i=1}^k{X}_i=n\right.\right) $$

The second fact is a relationship between conditional Poisson probabilities, and an expression involving the sum of truncated Poisson random variables. The following is a slight variant of a theorem due to Levin [4].

**Theorem 2** *Let* ${X}_i^{\left({a}_i,{b}_i\right)}$ *be a truncated Poisson random variable, with probability mass function*

$$ P\left({X}_i^{\left({a}_i,{b}_i\right)}=x\right)=P\left({X}_i=x|{a}_i<{X}_i\le {b}_i\right) $$

*where X*_*i*_ *is a Poisson random variable with rate λ*_*i*_*. For vectors a and b, let X*^(*a*, *b*)^ *be the vector containing all of these truncated Poisson random variables. Let E be the set of vectors x such that a*_*i*_ < *x*_*i*_ ≤ *b*_*i*_. *Then,*

$$ P\left(X\in E\left|\sum \limits_{i=1}^k{X}_i=n\right.\right)=\prod \limits_{i=1}^kP\left({a}_i<{X}_i\le {b}_i\right)\frac{P\left(\sum \limits_{i=1}^k{X}_i^{\left({a}_i,{b}_i\right)}=n\right)}{P\left(\sum \limits_{i=1}^k{X}_i=n\right)} $$

**Proof:** By Bayes’ theorem,

$$ P\left(X\in E\left|\sum \limits_{i=1}^k{X}_i=n\right.\right)=P\left(X\in E\right)\frac{P\left(\left.\sum \limits_{i=1}^k{X}_i=n\right|X\in E\right)}{P\left(\sum \limits_{i=1}^k{X}_i=n\right)} $$

Substituting $P\left(\sum \limits_{i=1}^k{X}_i^{\left({a}_i,{b}_i\right)}=n\right)$ for $P\left(\left.\sum \limits_{i=1}^k{X}_i=n\right|X\in E\right)$ and $\prod \limits_{i=1}^kP\left({a}_i<{X}_i\le {b}_i\right)$ for *P*(*X* ∈ *E*) yields the theorem. □

This theorem enables a fast calculation of the multinomial probability. The rate-limiting step is calculation of the probability distribution of $\sum \limits_{i=1}^k{X}_i^{\left({a}_i,{b}_i\right)}$. Levin [4] provided two suggestions for computing this probability distribution: the first by convolution of the distributions of each ${X}_i^{\left({a}_i,{b}_i\right)}$, and the second using an Edgeworth expansion of the probability distribution of $\sum \limits_{i=1}^k{X}_i^{\left({a}_i,{b}_i\right)}$. We implemented both suggestions, which are used for different values of *n*. For small values of *n*, convolution is performed, using The Fastest Fourier Transform In The West algorithm [5]. For large values of *n*, an Edgeworth expansion is used. However, whereas Levin [4] used the first four terms in the expansion, we continue adding terms until the last term added is sufficiently small.

SCOPIT also computes Bayesian posterior probability distributions for the multinomial probabilities. The multinomial probabilities described above are a function of the population frequencies. When the true population frequencies are not known, but observed frequencies from a previous experiment are available, SCOPIT computes a posterior distribution for the frequencies. The prior used for the frequencies is Dirichlet(0, …, 0), following Jaynes [6] for an experiment in which the possible outcomes are not known in advance. The resulting posterior is Dirichlet(*n*_1_, …, *n*_*k*_), where *n*_*i*_ is the number of cells observed from population *i*. Possible frequency vectors are randomly drawn from this posterior using the R package rBeta2009 [7, 8]. Then, the desired multinomial probability is calculated from each sampled frequency vector, resulting in samples from the posterior distribution of possible multinomial probabilities. A posterior distribution over the number of cells required is calculated in the same way.

## Results

### Estimating required sample size using the multinomial distribution

We make the simplifying assumption that a successful experiment requires sampling a sufficient number of representatives from each subpopulation of interest in the tissue. Defining *c* as the required number of representatives from each subpopulation, *N*_*i*_ as the number of cells of subpopulation *i* which are sampled, and *k* as the number of subpopulations of interest, then the probability of meeting this condition is

$$ P\left({N}_1\ge c,{N}_2\ge c,\dots, {N}_k\ge c\right) $$

Assuming that a fixed number of cells are chosen at random from the population, the distribution of *N*_1_, …, *N*_*k*_ is multinomial. To calculate this probability, we created an R implementation of a previously described algorithm [4], described further in the Implementation section. Our implementation is available for R scripting in the package “pmultinom”, available from CRAN (Table 1).

**Table 1:** Package functions for pmultinom. This table lists the R functions for the package “pmultinom” for calculating multinomial probabilities

Function	Arguments	Description
pmultinom	lower, upper, size, probs, method	Probability that a multinomial random vector is elementwise greater than “lower” and elementwise less than or equal to “upper”. “size” and “probs” specify the parameters of the multinomial distribution. Either “lower” or “upper” may be left unspecified.
invert.pmultinom	lower, upper, probs, target.prob, method	Returns the “size” parameter required for pmultinom to reach the target probability “target.prob”.

Our web tool, SCOPIT, provides an interactive interface for multinomial calculations. SCOPIT provides both prospective and retrospective calculations, described below.

### Prospective calculations

SCOPIT’s prospective mode is intended to estimate the number of cells that must be sampled in a single-cell sequencing experiment. Ideally, the number of cells can be decided by finding a number of cells, *n*^∗^, such that the above multinomial probability is above a specified success probability, *p*^∗^. Such a calculation would require specifying the frequency of each subpopulation of cells in the tissue, but the precise subpopulation frequencies are usually unknown before performing the experiment.

The strategy implemented in the prospective mode is to specify the frequency of the rarest subpopulations that the researcher intends to find, as well as *k*, the number of populations with approximately this frequency. Both numbers are relevant, since it is harder to find, for example, 10 subpopulations with frequency 1%, than it is to find only one.

The required number of cells is defined as follows:

$$ {n}^{\ast }=\min\;\left\{n\;|\;P\left({N}_1\ge c,{N}_2\ge c,\dots, {N}_k\ge c\right)\ge {p}^{\ast}\right\} $$

**Fig. 1:**
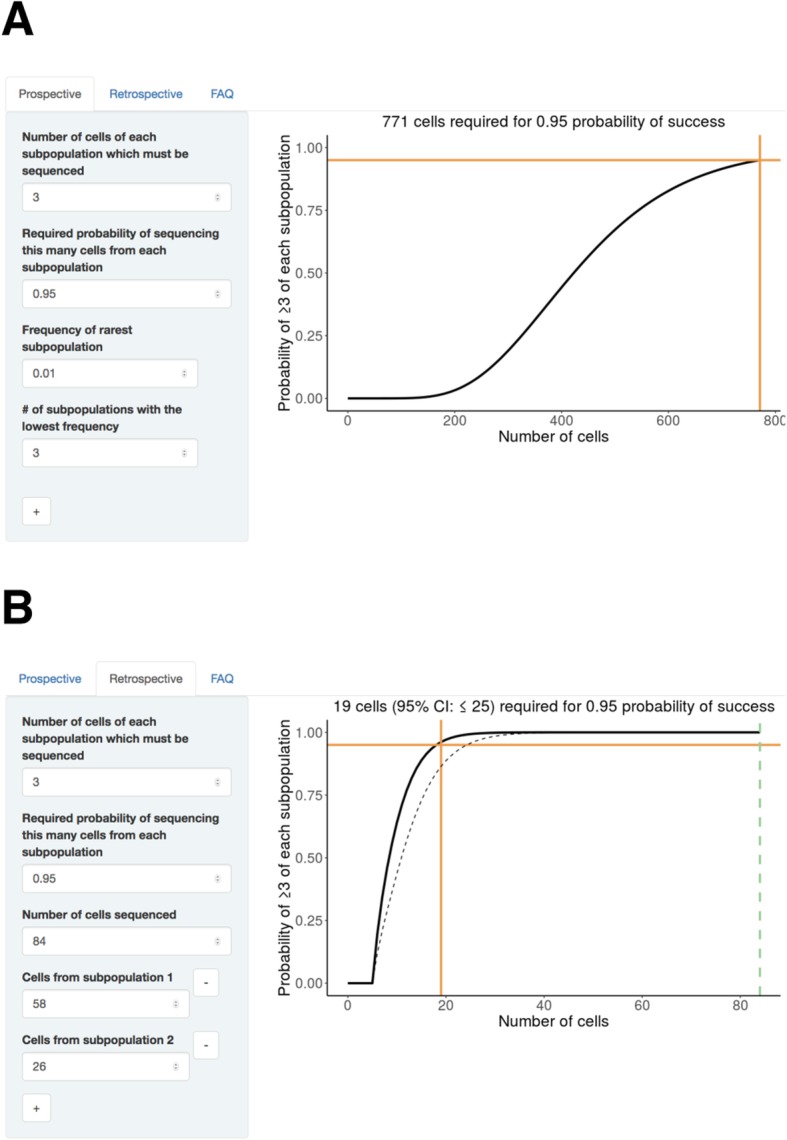
SCOPIT interface. **a**. Interface for prospective calculations. Orange lines identify the number of cells required and the target probability of detecting a specified number of each subpopulation. **b**. Interface for retrospective calculations. The number of cells which were sequenced is entered, and is marked on the plot with a dotted green line. In this example, the orange line is far to the left of the dotted green line, suggesting that more cells were sequenced than required to detect these three subpopulations. To quantify confidence in the results, a dotted black line is plotted that shows the lower end of a 95% credible interval for the probability. The plot title states the upper end of a 95% credible interval for the number of cells required

SCOPIT reports *n*^∗^ along with a plot of the probability as a function of the number of cells sequenced (Fig. 1a).

This mode requires only one subpopulation frequency to be specified: the minimum frequency among all subpopulations of interest. The SCOPIT interface does enable the user to add additional subpopulations with higher frequencies, but the user will find that these additional subpopulations have negligible effects on *n*^∗^, unless they are very close in frequency to the rarest subpopulations. This phenomenon justifies specifying only the lowest frequency.

### Retrospective calculations

After an experiment has been performed, estimates of the subpopulation frequencies are available as input parameters. It is then possible to use SCOPIT in retrospective mode to estimate how many cells would be required, in a hypothetical replicate experiment, to detect all *k* observed subpopulations, with *c* representatives from each. In retrospective mode, the information required from the user consists of the total number of cells sequenced in a previous experiment, and the number of cells observed from each subpopulation. With this information, SCOPIT will calculate, for each number of cells *n*, the probability *P*(*N*_1_ ≥ *c*, *N*_2_ ≥ *c*, …, *N*_*k*_ ≥ *c*), assuming the true subpopulation frequencies are equal to the empirically observed ones. For example, in Fig. 1b, we use single cell DNA data from a triple-negative breast tumor [9] in which the authors sequenced *N* = 84 single cells and detected two major clonal subpopulations. Using SCOPIT we estimated that only 19 cells were required to detect the two subpopulations with a 0.95 probability, suggesting that this study sequenced about 4 times the number of cells that were necessary.

Because the retrospective analysis involves uncertainty about the true frequencies of each population, SCOPIT provides measures of uncertainty using Bayesian credible intervals at a 95% confidence level. For the number of cells required, SCOPIT reports the upper end of a one-sided credible interval, which is interpretable as the highest number of cells consistent with the data. For the probability of obtaining a sufficient number of cells from each population, SCOPIT plots the lower end of a one-sided credible interval, interpretable as the lowest probability consistent with the data. In the example described above, the credible interval boundaries were close to the estimated values, indicating that the estimated values were strongly supported by the data provided.

The retrospective tool is useful for planning a second experiment, assuming that all the subpopulations of interest were observed in the first experiment, and that the underlying subpopulation frequencies are consistent in both experiments. Although the exact subpopulation frequencies are not known, overconfident conclusions on the basis of limited information can be avoided using the credible intervals provided by the retrospective tool.

### Comparison with independence approximation

Another previous software tool for estimating single cell sample sizes is an unpublished web application (https://satijalab.org/howmanycells). The previous tool is based upon two simplifying assumptions: that the subpopulations have equal frequencies, and that the observed frequencies of each subpopulation are statistically independent. Under these assumptions:

$$ P\left({N}_1\ge c,{N}_2\ge c,\dots, {N}_k\ge c\right)=P{\left(N\ge c\right)}^k $$

where *N* represents the number of cells sampled from an arbitrary subpopulation. To compare the independence approximation method to SCOPIT, the required number of cells was calculated with and without the independence assumption (Table 2). The calculations performed under the independence assumption underestimated the required number of cells by at most 1 cell and were highly similar. These data suggests that using independence approximation is an alternative approach that can also be used for estimating single cell sample sizes.

**Table 2:** Comparison of Independent Approximation and Exact Calculations.

Subpopulation frequency	# of subpopulations	Cells required (exact)	Cells required (approx.)
0.1	6	186	186
0.2	3	85	85
0.3	2	53	53
0.1	8	191	191
0.2	4	87	87
0.4	2	39	39
0.1	9	193	193
0.3	3	55	55
0.1	10	195	194
0.2	5	89	89
0.5	2	30	30

The number of cells required to achieve a 95% certainty of sampling sufficiently many cells from each subpopulation. The number of cells was calculated in two ways: by an exact calculation, and by an approximate calculation in which the counts of different subpopulations were assumed to be independent

## Discussion

SCOPIT’s function is to calculate the number of cells that must be sampled in a single-cell sequencing experiment, on the basis of input subpopulation frequencies, and under the assumption of random sampling. To achieve this goal, we implemented a fast multinomial probability calculation approach that is provided as open access software through the R package ‘pmultinom’. This method enables calculations at speeds sufficient for interactive plotting. The retrospective sample size calculation performed by SCOPIT is distinct from estimation of the number of undiscovered subpopulations [10] or the number likely to be discovered in further sampling [11], and can instead be interpreted as the required sample size of a replicate experiment which would detect the same subpopulations as the original experiment.

To determine the number of cells required, SCOPIT calculates the probability of sampling sufficiently many representatives of each subpopulation. The probability calculated by SCOPIT is relevant to a wide variety of analyses and technologies, but specific technologies introduce additional experimental design considerations. For example, in single-cell differential expression analysis, it is important not only to sample sufficiently many cells, but also to sample sufficiently many transcripts from each cell. Other tools have been developed to calculate the probability of detecting a specific transcript [12], to calculate the power to detect differential expression [13], and to determine the number of cells and reads required to find accurate low-dimensional representations of single-cell RNA sequencing data [14]. Accommodating the unique aspects of other technologies and analyses is an important topic for future research in the design of single-cell sequencing experiments.

A previous tool is available for calculating the number of cells to sequence (https://satijalab.org/howmanycells) and a direct comparison to SCOPIT shows that it generates results that are highly similar to SCOPIT, despite using independent approximations instead of exact probabilities. However SCOPIT offers several additional features, including the ability to enter multiple cell type frequencies, and interfaces to perform both prospective estimates of the sample sizes for planning experiments and retrospective calculations which include measures of confidence in the result.

While SCOPIT can be used to decide how many cells to sample from a tissue, another important question is how many spatial regions to sample to capture the diversity of the population. In the case of sampling from tumor tissue, the question of how widely to sample can be addressed by simulating the generation of intratumor heterogeneity [15], followed by simulating sampling. However, simpler statistical calculations which avoid detailed simulations are currently not available and represent an important future direction.

## Conclusions

This study reports a useful tool for estimating sample size calculations for planning single cell sequencing experiments prospectively and retrospectively. We expect that SCOPIT will have applications in many diverse areas of biology, and for planning experiments on a variety of single cell technologies (scDNA, scRNA and scATAC-seq).

## Availability and requirements

Project name: SCOPIT

Project homepage: https://github.com/navinlabcode/scopit

Web interface: http://www.navinlab.com/SCOPIT

Operating system: Platform independent

Programming language: R

License: AGPL v3

## Data Availability

The source code used in the current study are available from GitHub, https://github.com/navinlabcode/

## Abbreviations

SCOPIT: Single-cell one-sided probability interactive tool
